# First isolation of atypical enteropathogenic *Escherichia coli* from geese (*Anser anser domestica*) and first description of atypical EPEC from turkeys and pigeons in Hungary

**DOI:** 10.1186/s12917-021-02968-w

**Published:** 2021-08-05

**Authors:** András Adorján, Ákos Thuma, László Könyves, István Tóth

**Affiliations:** 1 Department of Microbiology and Infectious Diseases, University of Veterinary Medicine, Budapest, Hungary; 2 Veterinary Diagnostic Directorate, National Food Chain Safety Office, Budapest, Hungary; 3 Department of Animal Hygiene and Mobile Clinic, University of Veterinary Medicine, Budapest, Hungary; 4 Institute for Veterinary Medical Research, Centre for Agricultural Research, Budapest, Hungary

**Keywords:** Atypical enteropathogenic *﻿Escherichia coli*, aEPEC, Water fowl, Geese, Multidrug resistance

## Abstract

**Background:**

*Escherichia coli* is a bacterial species widely distributed among mammals and avian species, and also a member of the normal intestinal microbiota. However, some *E. coli* strains of different pathotypes can cause disease in both humans and animals. Atypical enteropathogenic *E. coli* (aEPEC) can infect both animals and humans or influence the severity of other ongoing infections.

**Results:**

In the present study, a total of 332 samples were collected from ducks, geese, turkeys, chickens, and pigeons from the Hungarian Veterinary Diagnostic Directorate, two slaughterhouses, two pigeon keepers and one backyard chicken farm. *E. coli* was isolated and verified from 319 samples. The isolates were screened by PCR for diarrheagenic *E. coli* pathotypes. Altogether seven atypical enteropathogenic *E. coli* (aEPEC) strains were identified: two from four-week-old dead turkeys, two from force-fed geese, and three from pigeons. No further pathotypes were identified in the collection. The atypical EPEC strains were classified phylogenetically to B1, B2, and F, and four out of the seven aEPEC isolates proved to be multidrug resistant. Serotypes of aEPEC strains were uniform collected from same farms and showed diversity between their origins with O76, O145, O109 serogroups.

**Conclusions:**

This is the first report in the literature about aEPEC in goose (*Anser anser domestica*). Furthermore, this is the first isolation of aEPEC from turkeys and pigeons in Hungary. The uneven distribution of aEPEC in different age groups of poultry suggests that aEPEC disappears with growing up, but stress (e.g.: force-feeding) and concurrent diseases might promote its reappearance in the intestine.

## Background

*E. coli* is a bacterial species widely distributed among mammals and birds. The majority of *E. coli* strains take part in maintaining the normal function of the healthy intestinal tract and protect it from invasion by pathogenic bacteria. However, certain *E. coli* strains can cause mild or more severe diseases as facultative pathogenic bacteria in animals and humans as well. *E. coli* strains are categorized into extraintestinal (ExPEC) and intestinal (DEC) pathogenic groups depending on the site of the infection caused by them. ExPEC strains are classified into three categories, namely uropathogenic *E. coli* (UPEC), meningitis-associated *E. coli* (MNEC), and avian pathogenic *E. coli* (APEC). All DEC infect mainly the intestinal tract, but the infection mechanism and process vary by pathotype. Therefore, DEC was divided into six pathogenic groups, namely enteropathogenic *E. coli* (EPEC), verotoxigenic/shigatoxigenic *E. coli* (VTEC/STEC), enterotoxigenic *E. coli* (ETEC), enteroinvasive *E. coli* (EIEC), enteroaggregative *E. coli* (EAEC), and diffusely adherent *E. coli* (DAEC). These pathotypes were identified on the basis of their key virulence factors (*eae*-EPEC, *eae* and *stx*-VTEC, *stA* and *lt1*-ETEC, *ipaH*-EIEC, *aggR*-EAEC) and their histological effects (DAEC) [1, 2].

*E. coli* is a common cause of human infection and diarrhea in the world [3, 4]. In such cases, poultry are an important source of human exposure because chickens, turkeys and waterfowl are kept in high numbers and their products are consumed in the largest volume in the world as a meat source. Wild birds and free-range poultry also have a high chance of spreading possibly pathogenic *E. coli* strains. Poultry carry pathogenic *E. coli* in their intestines [5, 6] or the bacteria may be present on poultry-derived products [7–9] in the priority order of EPEC, VTEC, ETEC, EIEC, and DAEC. EPEC is an important pathotype based upon the frequency of infections caused by it in humans both in the developing and the developed countries [3, 4], and sometimes it causes mass outbreaks [10].

EPEC was divided into typical EPEC (tEPEC) and atypical EPEC (aEPEC) according to the pilus (bundle-forming pilus, BFP) forming ability (encoded by *bfpA* gene and its EAF plasmid carrier) of the bacterium, which is missing from aEPEC strains [4, 11]. The frequency of typical EPEC in epidemics and diarrhea cases decreased in the last few decades, and this pathotype is harbored permanently only by humans. The role of tEPEC in human infections has been taken over by aEPEC. Atypical EPEC has increasing frequency in diarrhea cases. This position of aEPEC is promoted by its wide presence in several animal species including poultry, which can raise the possibility of zoonotic risk [3].

To date, there is little information about the effect of aEPEC on animal species. However, many studies have demonstrated that aEPEC can also cause diarrhea in different animal species and influence the outcome of these infections in dogs, cats, turkeys, and lambs [12–16]. Some authors have also suggested that these animal species could act as the source of human aEPEC infections [17, 18].

Broilers frequently harbor aEPEC strains and their meat can also carry this pathogenic *E. coli* after slaughter [7, 9, 19, 20]. However, so far we have only very limited information about the existence of aEPEC in waterfowl species and pigeons.

Therefore, our aim was to investigate the presence of DEC pathotypes in five common poultry species, mainly in waterfowl, and to determine the possible effect of age on aEPEC frequency.

## Results

### Bacterial strains

Overall, 332 swab samples were collected from poultry. Each sample came from one bird as an individual specimen. However, lactose-positive colonies were isolated only from 319 samples (*n* = 35 pigeons, *n* = 42 chickens, *n* = 87 ducks, *n* = 101 geese, *n* = 54 turkeys), and they were verified biochemically as *E. coli*. *Escherichia coli* strains originating from the Diagnostic Directorate (DD) came from a pigeon (*n* = 1), chickens (*n* = 29), ducks (*n* = 36), geese (*n* = 53), and turkeys (*n* = 4). *Escherichia coli* bacteria isolated at the Backyard (BY) from pigeons (*n* = 34) and chickens (*n* = 13). *Escherichia coli* were identified from ducks (*n* = 51), geese (*n* = 48) and turkeys (*n* = 50) from Slaughterhouse (SH) (Table 1).

**Table 1:** Age distribution of the samples collected and number of samples positive to *eae* gene

	Ages	Pigeon	Chicken	Duck	Goose	Turkey	Overall
**Diagnostic Directorate**	0-1 week		14	27			41
	1-6 weeks			4	26	^a^2/4	34
	7-16 weeks				13		13
	15 weeks			4			4
	17 weeks-6 months				3		3
	6 months	^a^1/1					1
	6-12 months				11		11
	Over 1 year		15	1			16
	Sum	**1**	**29**	**36**	**53**	**4**	**123**
**Homeyard**	Nestlings	^a^2/12					﻿12
	3-4 months	8					8
	6 months	4					4
	2-3 years	10	13				23
	Sum	**34**	**13**				**47**
**Slaughterhouse**	14 weeks			51			51
	16 weeks				^a^2/48		48
	20 weeks					50	50
	Sum			**51**	**48**	**50**	**149**
	**Total**	**35**	**42**	**87**	**101**	**54**	**319**

^a^n/n means: *eae* (intimin) positive sample(s)/all sample(s)

### Pathogenic groups

None of the *E. coli* isolates belonged to the VTEC, ETEC, EAEC and EIEC pathotypes because of the absence of *stx1* and *stx 2* (VTEC), *sta* and *lt1* (ETEC), *aggR* (EAEC), *ipaH* (EIEC) virulence genes screened by PCR [1]. In seven samples, the *eae* (encoding intimin adhesin) gene was detected, and thus these samples were identified as the EPEC pathotype [11]. We further classified EPEC strains as aEPEC on the basis of the missing EPEC Adherence Factor (EAF) plasmid and its carried *bfpA* gene by PCR [11]. All aEPEC isolates carried *tir* (translocated intimin receptor) which is a key virulence factor of EPEC and EHEC. Our aEPEC strains isolated from turkeys (*n* = 2 from the DD, both 4 weeks old), pigeons (*n* = 1 from the DD, 6 months old, *n* = 2 from BY, both nestlings) and geese (*n* = 2 from SH, both 16 weeks old).

### Phylogenetic, serogroups and antimicrobial resistance of the aEPEC isolates

Both of turkey aEPEC strains were MDR, but they represented different phylogenetic groups, namely B1 (O/not typable:H/not moving) and F (O76:H/not moving). One turkey aEPEC had an exceptional feature, showing resistance to 14 out of the 15 tested antimicrobials and being sensitive only to gentamicin. Both of the goose aEPEC strains were MDR and showed resistance to 9 and 11 antimicrobials, respectively. However, they belonged to the same phylogenetic and serogroup, B2 and O145:H(spontaneous agglutination) respectively. Pigeon aEPEC strains belonged to the B1 phylogenetic group. However, pigeon aEPEC strains were resistant against maximum four antimicrobials and one strain showed resistance to only two. Nestling pigeons originated from one farm and has same serotype (O109:H21). Atypical EPEC from 6 month old pigeon serotype was O(not typable):H35 (Table 2).

**Table 2:** Antimicrobial resistance patterns, phylogenetic and serogroups of aEPEC isolates

Species	Age	ECOR	Serotype	Antibiotic resistance pattern
Turkey	4 weeks	B1	O(NT):H(NM)	AMC, AMP, CHL, CIP, ENR, FOX, KAN, NAL, NIT, SMX, STR, SXT, TET, TMP
Turkey	4 weeks	F	O76:H(NM)	AMC, AMP, CHL, CIP, ENR, NAL, SMX, STR, SXT, TET, TMP
Goose	16 weeks	B2	O145:H(SP)	AMC, AMP, NAL, NIT, SMX, STR, SXT, TET, TMP
Goose	16 weeks	B2	O145:H(SP)	AMC, AMP, CIP, ENR, NAL, NIT, SMX, STR, SXT, TET, TMP
Pigeon	nestlings	B1	O109:H21	AMP, NIT, SMX
Pigeon	nestlings	B1	O109:H21	AMC, SMX
Pigeon	6 months	B1	O(NT):H35	AMC, AMP, SMX, STR

*Abbreviations*: *ECOR* Phylogenetic groups, S*erotype: NT* not typable, *NM* not moving, *SP* spontaneous agglutination, *AMC* amoxicillin, *AMP* ampicillin, *CHL* chloramphenicol, *CIP* ciprofloxacin, *ENR* enrofloxacin, *FOX* cefoxitin, *KAN* kanamycin, *NAL* nalidixic acid, *NIT* nitrofurantoin, *SMX* sulphonamide, *STR* streptomycin, *SXT* sulphonamide + trimethoprim, *TET* tetracycline, *TMP* trimethoprim

The prevalence of antimicrobial resistant aEPEC strains isolated from turkeys and geese was significantly (*p* = 0.0037) higher than that found in pigeons.

## Discussion

Because of the scarcity of relevant information in the literature, our aim was to study the distribution of aEPEC in five important poultry species and the possible effects of age on its prevalence.

Several research groups have reported the high prevalence of aEPEC around slaughtering age in broilers (at 5–6 weeks of age) and on their carcass [5–7, 9, 19]. Furthermore, some authors have suggested that aEPEC strains present a potential risk of zoonosis [21–23]. However, we were curious about the presence of aEPEC in different age groups of chickens. We did not find atypical EPEC in young chicks (*n* = 14 from 3 farms) and adult chickens (*n* = 28 from 6 farms), although we could have presumed this from our previous studies and from the findings of other authors [7, 9, 19].

There was no high caseload of dead turkeys (*n* = 4 from one farm) at the DD in 2020, but two aEPEC strains were isolated from two four-week-old turkeys. This finding was not unique, as aEPEC had been reported previously in turkeys [24] and found to be associated with other co-infections [14, 25]. Atypical EPEC was not detected by us from the slaughterhouse samples (*n* = 50), where the age of turkeys was around 20 weeks.

Results had been very scarce about the prevalence of aEPEC in ducks [26], and no data were available about aEPEC in geese yet. Atypical EPEC were not carried by ducks (*n* = 87 from 9 farms) according to our findings, which were in harmony with the results of another research group [27]. However, our samples cannot be compared properly with the results of others, because the other studies did not focus on or record the ducks’ age. Our *E. coli* strains came from young (0–1 week old, *n* = 31), middle-aged (14–15 weeks old, *n* = 55) groups, and in one case the age was over one year. Furthermore, our two aEPEC strains isolated from geese represented the first detection of aEPEC in this species. Interestingly, they were isolated from the middle-aged group, from force-fed geese used for *foie gras* production.

Atypical EPEC were carried by 3 pigeons (*n* = 1 from the DD, *n* = 2 from BY), one of which originated from a 6-month-old pigeon and two from nestling pigeons. We could not detect atypical EPEC in older and adult pigeons. Our findings in pigeons are in harmony with the results of other scientist in that pigeons can carry aEPEC. However, the comparison with the findings of other researchers was very limited because they focused on searching antibiotic resistance and virulence genes of *E. coli* and did not record the age of sampled pigeons which may influence *E. coli* pathogroups distribution [26, 28–30].

 In summary, according to our own findings and data of the literature about the distribution of aEPEC in the main poultry species, we suppose that all poultry have the capability to carry aEPEC. However, we suppose that the age of the birds and certain environmental factors (e.g.: force-feeding) or diseases (causing mortality in our cases) can influence the prevalence of carriage. We assume that poultry do not carry aEPEC in a considerable degree in the first weeks of life, and only in the later phases, around 4–6 weeks of age, can aEPEC propagate in high numbers in the intestines of healthy [9, 19] and sick birds [14]. Later on aEPEC will disappear from poultry as recorded by others in sheep [13].

By studying the antimicrobial resistance of aEPEC, we found significant differences between turkeys, geese and pigeons. Turkeys and geese as intensively kept birds had more opportunity to get medical treatment from time to time. This fact could be behind the very high levels of antimicrobial resistance found in turkey and goose. However, pigeons, especially as nestlings, have a lower chance to receive antimicrobial treatment, and thus the members of their microbiota have lower resistance to antimicrobials. However, the evidence that aEPEC strains are frequently MDR, especially against widely used antimicrobials, can suggest a possible horizontal gene transfer of resistance genes to humans as well.

Three out of the 7 aEPEC strains belonged to phylogenetic groups F and B2, which contain potential ExPEC strains and, therefore, could pose a higher risk of zoonotic infection. Furthermore, groups F and B2 (both of which had belonged to the B2 phylogenetic group earlier) are common among aEPEC strains as we detected earlier [19]. The serotypes of aEPEC strains were uniform from same farms and showed diversity (O76, O145, O109) comparing their origins.

## Conclusions

In summary, our main result is to report the presence of aEPEC in goose (*Anser anser domesticus*) for the first time in the literature. Furthermore, we first isolated aEPEC from turkeys and pigeons in Hungary. From the uneven distribution of aEPEC in the different age groups of poultry we conclude that aEPEC disappears with the advancement of age.

## Methods

### Sample collection

Samples were collected from poultry carcasses at the Veterinary Diagnostic Directorate of the National Food Chain Safety Office (DD; Budapest, Hungary) from sick birds (animals originated from 8 chicken, 8 duck, 10 goose, 1 turkey farm) and from healthy poultry at two slaughterhouses (SH) (one waterfowl and one turkey), at one backyard chicken farm (BY) and at two pigeon keepers (PK) in 2020.

We collected samples from birds of diverse ages (from day-old to 3 years) in order to identify possible differences in the distribution of the *E. coli* pathotypes. Birds were classified into age groups for better visualization of the age distribution in each poultry species.

Samples were aseptically collected from the cecum of dead or slaughtered birds and from the cloaca of live chickens and pigeons with a sterile cotton swab, and they were stored at 4 °C at most for 2 h before further processing.

### Bacteriological identification

All cotton swabs were smeared on MacConkey agar, and one lactose-positive colony from each sample was inoculated further until they seemed to be uniform. Then, bacterial colonies were examined by primary (catalase, oxidase) and secondary biochemical tests (indol, methyl red, Voges–Proskauer, citrate utilization tests) to confirm them as *E. coli*. Their pure cultures were kept at – 80 °C for long-term storage.

### Antimicrobial resistance

Antimicrobial resistance of the bacteria was determined using the disc diffusion method performed according to the recommendations of the Clinical and Laboratory Standards Institute (M100-S25, 2020) [31]. Briefly, the procedure was as follows: 0.5 McFarland even solutions were made from pure bacterial cultures and were streaked evenly on Mueller–Hinton agar. Then, the antimicrobial discs were evenly placed on it and the plates were incubated overnight at 37 °C until their evaluation. Based on the appearing inhibition zones, the bacteria were categorized into a resistant or a sensitive group (the intermediate group was regarded as sensitive) according to the CLSI recommendation for the *Enterobacteriaceae* family [31, 32].

The following antimicrobials were used: penicillins [ampicillin (10 µg)]; ß-lactam/ß-lactam inhibitor combination [amoxicillin-clavulanate (20 µg/10 µg)]; cephems [cefoxitin (30 µg)]; aminoglycosides [gentamicin (10 µg), kanamycin (30 µg), streptomycin (10 µg)]; tetracyclines [oxytetracycline (30 µg)]; fluoroquinolones [ciprofloxacin (5 µg), enrofloxacin (5 µg)]; quinolones [nalidixic acid (30 µg)]; folate pathway inhibitors [trimethoprim (30 µg), sulfonamide (300 µg), trimethoprim + sulfonamide (1.25 µg/23.75 µg)]; phenicols [chloramphenicol (30 µg)]; nitrofurans [nitrofurantoin (300 µg)]. If an *E. coli* strain showed resistance to more than four groups of antimicrobials, we considered it a multidrug-resistant strain (MDR).

### Genotypic evaluation of *Escherichia coli*

DNA templates were made from *E. coli* by the boiling method. In this procedure we inoculated 2 ml LB (Luria–Bertani) medium with the pure culture of isolated *E. coli* and incubated the culture overnight at 37 °C. In the next step five hundred microliters bacterial broth was measured and centrifuged at 9000 rpm for 2 min, then the supernatant was discarded. The remaining pellets were covered with bi-distilled water and boiled for 10 min at 96 °C, then they were centrifuged for 10 s. The supernatants were removed as template and were used for further procedures.

The PCR master mix was made from DreamTaq Green© and its buffer according to the manufacturer’s recommendations (Invitrogen) with 0.5 µM specific primer for each reaction. The details of the primers used are summarized in Table 3.

**Table 3:** List of the primers used with their details and references

Gene	Primer name and its sequence (5’-3’)	Reference
*eae*	B52: AGGCTTCGTCACAGTTG	[33]
	B53: CCATCGTCACCAGAGGA	
*stx 1*	B54: AGAGCGATGTTACGGTTTG	[33]
	B55: TTGCCCCCAGAGTGGATG	
*stx 2*	B56: TGGGTTTTTCTTCGGTATC	[33]
	B57: GACATTCTGGTTGACTCTCTT	
*sta*	STa-F: TTTATTTCTGTATTGTCTTT	[34]
	STa-R: ATTACAACACAGTTCACAG	
*lt1*	LT1-F: AGCAGGTTTCCCACCGGATCACCA	[34]
	LT1-R: GTGCTCAGATTCTGGGTCTC	
*ipaH*	IPAH III: GTTCCTTGACCGCCTTTCCGATACCGTC	[35]
	IPAH IV: GCCGGTCAGCCACCCTCTGAGAGTAC	
*aggR*	aggR-3: CATCTCTTTGATAAGTCCTTCTCG	[36]
	aggRks-1: GTATACACAAAAGAAGGAAGC	
*bfpA*	EP1: AATGGTGCTTGCGCTTGCTGC	[37]
	EP2: GCCGCTTTATCCAACCTGGTA	
*eaf*	Eaf1: CAGGGTAAAAGAAAGATGATAA	[38]
	Eaf2: TATGGGGACCATGTATTATCA	
*chuA*	ChuA.1b: ATGGTACCGGACGAACCAAC	[39]
	chuA.2: TGCCGCCAGTACCAAAGACA	
*YjaA*	yjaA.1b: CAAACGTGAAGTGTCAGGAG	[39]
	yjaA.2b: AATGCGTTCCTCAACCTGTG	
*TspE4C2*	TspE4C2.1b: CACTATTCGTAAGGTCATCC	[39]
	TspE4C2.2b: AGTTTATCGCTGCGGGTCGC	
*arpA*	AceK.f: AACGCTATTCGCCAGCTTGC	[39]
	ArpA1.r: TCTCCCCATACCGTACGCTA	
*tir*	TirY474-F: CATATTTATGATGAGGTCGCTC	[40]
	TirS478-F: TCTGTTCAGAATATGGGGAATA	
	Tir-R: TAAAAGTTCAGATCTTGATGACAT	

Amplicons were separated by gel electrophoresis in 1.5 % gel at constant 110 V for approximately 30 min by the use of positive (amplicon of strain which carrying the appropriate gene) and negative control (empty PCR master mix) beside a 100-bp marker (Invitrogen©) for each run. The gels were recorded by the use of UV light with a camera.

### Phylogenetic classification

Phylogenetic groups of *E. coli* were determined by multiplex PCR (*﻿ChuA*, *﻿YjaA*, *﻿TspE4C2*, *﻿arpA*) described by Clermont et al. [39].

### Serotyping the *eae* positive *E. coli* strains

Determination of O and H antigens was performed with agglutination test described by Ørskov et al. [41] at the National Public Health Center, Budapest, Hungary.

### Statistical analysis

The comparison of frequency of antimicrobial resistance between *eae* positive strains was made with ANOVA (with 95 % confidence intervals) using the R statistical program (R Core Team, 2020) [42]. The other results were not as comprehensive as to require statistical tests for their comparison and interpretation.

## Data Availability

The datasets used and/or analysed during the current study are available from the corresponding author on reasonable request.

## Abbreviations

aEPEC: Atypical enteropathogenic *Escherichia coli*
BFP: Bundle-forming pilus
BY: Backyard chicken farm
DAEC: Diffusely adherent *Escherichia coli*
DD: Veterinary Diagnostic Directorate of the National Food Chain Safety Office
DEC: Intestinal pathogenic *Escherichia coli*
EAEC: Enteroaggregative *Escherichia coli*
EAF: EPEC adherence factor
EIEC: Enteroinvasive *Escherichia coli*
EPEC: Enteropathogenic *Escherichia coli*
ETEC: Enterotoxigenic *Escherichia coli*
ExPEC: Extraintestinal pathogenic *Escherichia coli*
MDR: Multidrug-resistant
MNEC: Meningitis-associated *Escherichia coli*
NT: Not typable
PCR: Polymerase chain reaction
SP: Spontaneous agglutination
SH: Slaughterhouse
STEC: Shigatoxigenic *Escherichia coli*
tEPEC: Typical enteropathogenic *Escherichia coli*
UPEC: Uropathogenic *Escherichia coli*
VTEC: Verotoxigenic *Escherichia coli*
